# Insulin amyloid polymorphs: implications for iatrogenic cytotoxicity†

**DOI:** 10.1039/d0ra07742a

**Published:** 2020-10-12

**Authors:** Keisuke Yuzu, Mikael Lindgren, Sofie Nyström, Jun Zhang, Wakako Mori, Risako Kunitomi, Terumasa Nagase, Keiichi Iwaya, Per Hammarström, Tamotsu Zako

**Affiliations:** Department of Chemistry and Biology, Graduate School of Science and Engineering, Ehime University 2-5, Bunkyo-cho Matsuyama Ehime 790-8577 Japan zako.tamotsu.us@ehime-u.ac.jp; Department of Physics, Faculty of Natural Sciences, Norwegian University of Science and Technology NO-7491 Trondheim Norway; IFM Chemistry, Linköping University SE-58183 Linköping Sweden; Department of Metabolism and Endocrinology, Tokyo Medical University Ibaraki Medical Center Ibaraki 300-0395 Japan; Department of Pathology, Sasaki Institute, Kyoundo Hospital Tokyo 101-0062 Japan

## Abstract

Amyloid specific fluorescent probes are becoming an important tool for studies of disease progression and conformational polymorphisms in diseases related to protein misfolding and aggregation such as localized and systemic amyloidosis. Herein, it is demonstrated that using the amyloid specific fluorescent probes pFTAA and benzostyryl capped benzothiadiazole BTD21, structural polymorphisms of insulin amyloids are imaged in localized insulin-derived amyloid aggregates formed at subcutaneous insulin-injection sites in patients with diabetes. It is also found that pFTAA and BTD21 could discriminate structural polymorphisms of insulin amyloids, so called fibrils and filaments, formed *in vitro*. In addition, it is shown that insulin drug preparations used for treating diabetes formed various types of amyloid aggregates that can be assessed and quantified using pFTAA and BTD21. Interestingly, incubated pFTAA-positive insulin preparation aggregates show cytotoxicity while BTD21-positive aggregates are less toxic. From these observations, a variety of amyloid polymorphic structures with different cytotoxicities formed both *in vivo* and *in vitro* by various insulin preparations are proposed.

## Abstract 2

Structural polymorphism of insulin amyloids *in vivo* can be recognized using novel amyloid specific fluorescent probes, pFTAA and BTD21.

## Graphical abstract

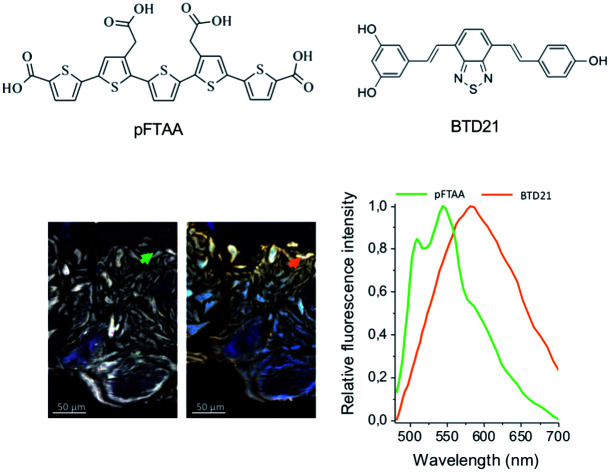

## Introduction

Alzheimer's disease (AD) and many other neurodegenerative disorders along with systemic amyloidosis, are associated with the accumulation of soluble and insoluble protein aggregates that are central to their pathogenesis.^1^ Primary amyloidosis is associated with faulty cell functions whereas reactive/induced (secondary) amyloidosis occurs as a complication of inflammation or some other tissue-degradation. In the treatment of diabetes mellitus, the goal is to achieve blood glucose levels closer to the normal range by subcutaneous injections of insulin preparations. Various insulin types including human insulin and its analogues, such as those with different point mutations and modifications, are used to alter the absorption of the drug as well as change solubility and shelf life.^2^ However, such treatment can give rise to iatrogenic fibrillar amyloidosis at the injection sites through the formation of insulin-derived amyloidosis or ‘insulin balls’, which cause poor glycemic control owing to impairments in insulin absorption.^3,4^ A recent case report on type 2 diabetes patients with insulin therapy also showed that some insulin balls may be cytotoxic, but others are not, implying amyloid polymorphism.^5^ Previously, we showed that bovine insulin produces two types of amyloids under different solvent conditions, filaments formed in the presence of a reducing reagent and fibrils formed from intact insulin, and that filaments are less toxic while fibrils are toxic.^6,7^ It has also been shown that insulin preparations, including human insulin and insulin analogues, formed amyloids *in vitro* in acidic conditions and elevated temperatures.^8^ Insulin amyloid polymorphism can also vary as a function on protein concentrations.^9^ However, the detailed structure and cytotoxicity of *in vivo*- and *in vitro*-formed amyloids derived from insulin preparations remains unclear. Thus, there is in this context a challenge to identify the structure and physiological phenotype of the various states of aggregating insulin.

The results in recent years of studies of advanced structure determination based on solid state NMR and cryo-EM imaging have pointed to the importance of the polymorphism of the related amyloid structures (*i.e.*, only certain folds/aggregates formed under quite different conditions), to pathogenesis.^10^ Although such structural characterization methods are very precise, they require substantial amounts of protein material to carry out the analysis. Moreover, these techniques are difficult to apply to living models and humans, and alternative highly sensitive methods are being sought based on fluorescence probes using microscopic and microprobe analyses. Because diabetes mellitus is very common and insulin preparations are widely used, there is interest in understanding these underlying mechanisms in further detail.

Previously, we showed that pFTAA and related luminescent conjugated oligothiophenes (LCOs) can be used to assess the fibrillation types of insulin.^11,12^ Although thioflavin T (ThT) fluorescence has been used to estimate the rate and the extent of fibril formation, these new dyes have some advantages over ThT: (1) these dyes can detect amyloid aggregates that show weak ThT fluorescence;^7,13^ and (2) information about the amyloid structure can be obtained.^14,15^ It was also recently shown that several variants of fluorescent phenolic styrylbenzo[*c*]-1,2,5-thiadiazoles, a new fluorescent amyloid probe based on benzothiadiazole (BTD) stilbene, can be used for spectral imaging of different amyloid-β (Aβ) fibril deposits.^16^

In this study, we demonstrated that the less toxic and flexible insulin filaments can be recognized with BTD21,^16^ a BTD stilbene ligand. Based on pFTAA and BTD21 staining and hyperspectral microscopy of patient-derived tissue sections of iatrogenic localized insulin amyloidosis (*i.e.*, insulin balls), we demonstrated that a variety of polymorphic amyloid aggregates, such as insulin fibrils and filaments,^6,7^ were co-localized in the tissue sections. Furthermore, pFTAA and BTD21 can also be used to classify a series of insulin preparations with respect to their propensity of forming one or the other of the archetypal amyloid types, fibrils or filaments. The results of TEM observation and cytotoxic assay also revealed that needle-like insulin fibrils detected by pFTAA showed cytotoxicity, whereas less ordered insulin filaments detected by BTD21 were less toxic. From these observations, a variety of amyloid polymorphic structures with different cytotoxicities formed both *in vivo* and *in vitro* by various insulin preparations are proposed.

## Materials and methods

### Patient tissue sections for hyperspectral imaging

Patient X with a 20 year history of type 1 diabetes has been previously reported.^4^ The patient had been using insulin lispro and insulin glargine I before the insulin ball was resected. Patient Y with a 9 year history of type 1 diabetes had developed insulin balls in the bilateral lower abdomen. The insulin ball used in this study was resected from the right lower abdomen about 4 years after the initial presentation of the insulin balls. Before this insulin ball was resected, the patient had been using many types of insulin preparations, including insulin aspart and insulin glargine I as the main ones. The following insulin preparations were also used: insulin aspart, insulin detemir, insulin degludec, insulin lispro and concentrated insulin glargine I.

The resected insulin ball was trimmed by the pathologist for each case. One of the pieces, which included the skin and insulin ball, was fixed for 6 h using 10% buffered formalin and then processed into a paraffin embedded block. Then, 4 μm sections of the block were deparaffinized by incubating them in xylene for 5 min, washing with distilled water, then they were stained with pFTAA and BTD21 as described.^12^ pFTAA and BTD21 were synthesized as described.^15,16^ This study was approved by the Ethics Committee of Tokyo Medical University Ibaraki Medical Center. The ethics committee approval number is 10–40. Written, informed consent was obtained from all participants in this study. The related experiments were performed in accordance with the Outline of Ethical Guidelines for Medical and Health Research Involving Human Subjects by the Ministry of Health, Labour and Welfare of Japan.

### Hyperspectral imaging

Hyperspectral microscopic imaging was performed using a Leica DM6000 B fluorescence microscope (Leica, Wetzlar, Germany) equipped with a SpectraCube module (Applied Spectral Imaging, Carlsbad, CA, USA) on samples as prepared in “Patient tissue sections for hyperspectral imaging” and “Preparation of aggregates from insulin preparations”. A 200-lumen lamp (Prior Scientific, Cambridge, UK) was used in conjunction with a filter cube (436/20 (LP460), Chroma Technology Corporation, Bellows Falls, VT, USA) to achieve the desired excitation and emission wavelengths. A 436 nm excitation filter was used, and hyperspectral images of 480–700 nm were collected, from which suitable ranges were selected for further analysis and plots. A long pass filter was used to block unwanted scattered excitation light.

### Preparation of aggregates from insulin preparations

The following insulin preparations were used: human insulin (Humulin N® [human I], Eli Lilly Japan, Kobe, Japan; Novolin R® [human II], Novo Nordisk Pharma, Tokyo, Japan), insulin lispro (Humalog®, Eli Lilly Japan), insulin aspart (NovoRapid®, Novo Nordisk Pharma), insulin glulisine (Apidra®, Sanofi, Tokyo, Japan), insulin glargine (Lantus® [glargine I], Sanofi, and Glargine® [glargine II], Eli Lilly Japan), insulin detemir (Levemir®, Novo Nordisk Pharma), and insulin degludec (Tresiba®, Novo Nordisk Pharma). All preparations were supplied as solutions. The properties of these insulin preparation are summarized in Table 1. Aggregation was induced by incubating the insulin solutions (4 mg mL^−1^) at 60 °C for 48 h without agitation.

**Table 1:** Insulin preparations used in this study

Insulin preparations	Type	Amino acids mutation	Modification
Human insulin	Regular	—	—
Insulin aspart	Rapid-acting	P28D (B chain)	—
Insulin degludec	Long-acting	T30 (B chain) was removed	16-Carbon fatty acid has been added to K29 (B chain) through a glutamic acid spacer
Insulin detemir	Long-acting	T30 (B chain) was removed	14-Carbon myristoyl fatty acid has been added to K29 (B chain)
Insulin glargine	Long-acting	N21G (A chain), K31R & T30R (B chain)	—
Insulin glulisine	Rapid-acting	K29E	—
Insulin lispro	Rapid-acting	P28K & K29P (B chain)	—

Bovine insulin fibrils and filaments were prepared for reference as described.^6^ In brief, bovine insulin was dissolved at a concentration of 20 mg mL^−1^ in 40 mM HCl (pH 1.5), then immediately diluted in fibril formation buffer (20% acetic acid, 100 mM NaCl, pH 1.6) or filament formation buffer (20% acetic acid, 100 mM NaCl, pH 1.6, 20 mM tris(2-carboxyethyl)phosphine (TCEP)) at a protein concentration of 2 mg mL^−1^ (345 μM). Aggregation was induced by incubating the insulin solution at 60 °C for 16 h without agitation. Samples were dialyzed by distilled water. The concentrations of fibrils and filaments were determined by subtracting the insulin monomer concentration measured in supernatants using an absorption coefficient of 1.0 for 1.0 mg mL^−1^ at 276 nm after centrifugation of a part of the incubated insulin samples at 15 000 rpm for 30 min.

For hyperspectral imaging, insulin amyloid aggregates formed *in vitro* were stained by pFTAA and BTD21. The sample solutions which contain 50 μM of insulin and 1.5 μM of probe in PBS (pH 7.4) and 8 mM HCl (pH 2.3) were prepared. These conditions were optimized according to the previous reports.^12,13,16^ Then, the sample solutions were centrifuged at 15 000 rpm for 30 min and the supernatant was removed. Afterwards, 5 μL of droplets were placed on the microscopy glass and covered with a cover glass.

### Fluorescence spectroscopy

The fluorescence of insulin amyloid fibrils was observed in the presence of ThT, pFTAA and BTD21 under neutral or low pH. For fluorescence measurements, sample solutions, which contain 5 μM of insulin samples and 1 μM of probe in PBS (pH 7.4) or 8 mM HCl (pH 2.3) were prepared. Fluorescence spectra were recorded using a Tecan Safire2 microplate reader. The excitation wavelength was typically 470 nm and bandwidth for excitation and emission was set to typically 10 and 5 nm, respectively.

### Transmission electron microscopy

Samples (5 μL each) were placed on carbon coated copper grids (Carbon-B, Ted Pella Inc.) and were incubated for 2 min. Excessive salt was removed with one wash of 5 μL deionized water and the grids were negatively stained with 2% uranyl acetate for 30 s. Grids were blotted dry and air dried overnight. Transmission electron microscopy (TEM) imaging was performed using a Jeol 1230 microscope operating at 100 kV and equipped with a Gatan digital camera.

### Toxicity assay

The cytotoxicity of the insulin samples was estimated by measuring cell viability after addition of insulin samples using an MTT cell proliferation kit as described.^6,7^ PC12 cells were maintained in RPMI1640 medium with 10% horse serum, 5% fetal bovine serum, penicillin (100 U mL^−1^), and streptomycin (100 μg mL^−1^) at 37 °C in 5% CO_2_. HeLa cells were maintained in MEM medium with 10% fetal bovine serum, penicillin (100 U mL^−1^), and streptomycin (100 μg mL^−1^) at 37 °C in 5% CO_2_. PC12 and HeLa cells were plated at a density of 20 000 cells per well and grown overnight. The cells were then incubated in 100 μL medium in the absence and presence of insulin samples diluted with PBS to various concentrations. After a 24 h incubation, 10 μL of MTT reagents were added each well. The cells were further incubated at 37 °C for 4 h in 5% CO_2_. The reaction was stopped by adding 100 μL of solubilization solution (10% SDS, 10 mM HCl). The plates were read with microplate reader at 562 nm. Each sample was assayed in triplicate, and the data were the average of three wells.

## Results and discussions

### Tissue sections from patients with insulin ball

**Fig. 1:**
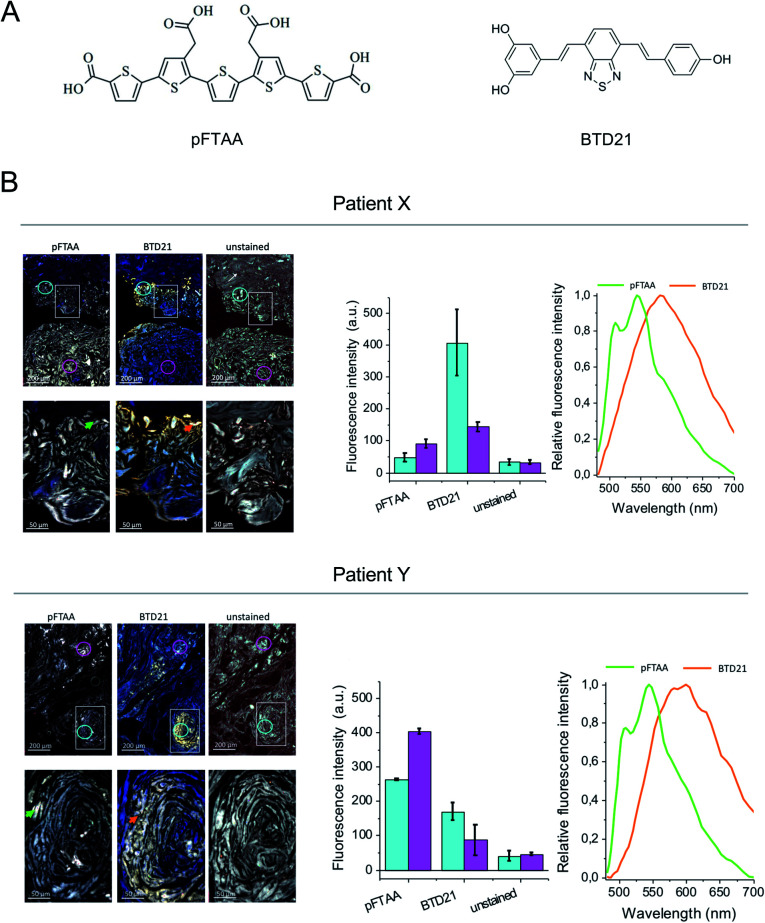
Hyperspectral fluorescence images of the patients' tissue sections stained with pFTAA and BTD21. (A) Structure of pFTAA and BTD21. (B) Hyperspectral fluorescence images of tissue sections from patients X and Y stained with pFTAA and BTD21. The upper panel shows an overview with 5× magnification. Close up images at a 20× magnification of a square are shown in the lower panel. Unstained sections are shown as the controls. Middle, fluorescent intensity of the ROI in two regions as indicated by color coding in each 5× magnification image (upper panels). The averaged values of >350 pixels in the area of the colored circles (cyan and pink) are shown. Right, normalized spectra at the indicated ROI as arrows in the 20× magnification images (lower panels) (green, pFTAA; orange, BTD21). The excitation wavelength was 436 nm.

The amyloid fluorescent probe pFTAA has been widely used for many types of fibrillar protein aggregates.^12^ It has been shown that specific ion bonds can form between the anionic carboxylate groups of pFTAA with regioregular lysine cationic side chains in amyloid fibrils which outline the periphery of the binding pocket of a Congo-red like binding site in a fibril model structure.^17^ More recently, several variants of fluorescent BTDs were developed for identifying amyloid polymorphs.^16^ The BTD21 compound which lacks the carboxylate groups likely binds in a different binding pocket than Congo red on Aβ fibrils in Alzheimer's disease mouse model brain.^16^ Here, we first examined the binding of pFTAA and BTD21 (chemical structures shown in Fig. 1A) to insulin fibrils and filaments (Fig. S1A and B†). Although native insulin with pFTAA or BTD21 did not show significant fluorescence, insulin fibrils with pFTAA showed high fluorescence under a neutral pH (pH 7.4) whereas insulin filaments with BTD21 showed high fluorescence in an acidic condition (pH 2.3), indicating that these two types of insulin amyloids can be discriminated using pFTAA and BTD21.

Iatrogenic insulin aggregates can cause issues for patients undergoing treatment. In this context sections of human insulin ball tissue specimens were investigated to delineate if conformational differences, *i.e.* fibrils *vs.* filaments, could be detected in the accumulated amyloid deposits. In this study, we performed hyperspectral microscopic imaging using pFTAA and BTD21 against consecutive sections from two patients treated with various insulin preparations (Fig. 1B). As a result, both stained tissue sections exhibited significant pFTAA and BTD21 fluorescence signals compared with the unstained sections, and the obtained fluorescence spectra showed similar spectral shapes to the spectroscopic data (Fig. S1A and B†). This suggests that accumulated amyloid in the insulin ball tissue could be detected with pFTAA and BTD21. Importantly, this result also suggests that structural polymorphism formed in the amyloid deposits, as would be expected if the fibril- and filament-type aggregates were deposited together during the same period. Intriguingly, there was a difference in staining patterns of pFTAA and BTD21 in the two tissue sections; when the intensity of BTD21 was low, the intensity of pFTAA staining was high, and *vice versa*. It was noticed that BTD21-positive amyloids were highly present in patient X, but in patient Y, more pFTAA-positive amyloids were detected. These observations demonstrate that insulin balls are composed of a variety of polymorphic amyloid aggregates.

### Estimation of amyloid formation from insulin preparations

**Fig. 2:**
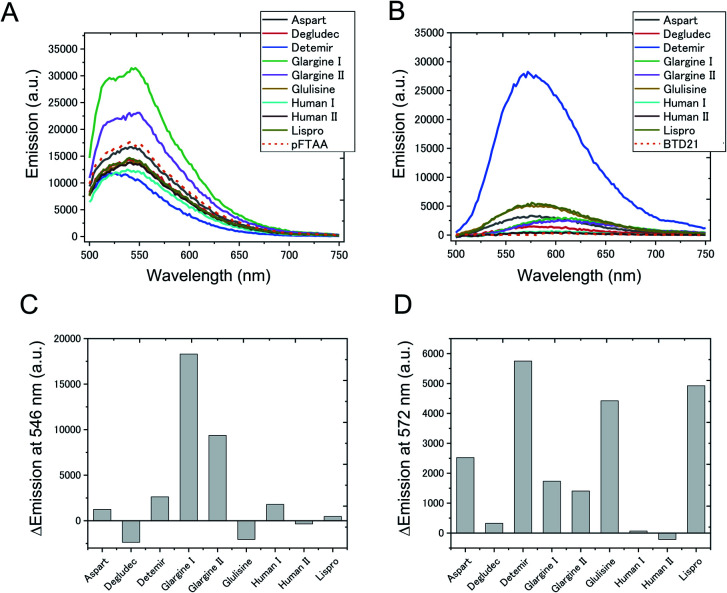
Fluorescence signal of (A) pFTAA and (B) BTD21 with various insulin amyloid preparations used for clinical use. In (C) and (D), the spectral intensity at the emission maximum is plotted after subtracting the corresponding signal obtained by staining the native (untreated) form of the insulin preparation. pFTAA shown at 546 nm, and BTD21 shown at 572 nm.

To confirm whether amyloid aggregates could be formed in incubated insulin drug preparations (Table 1), and to determine any variation in amyloid structure, we measured the pFTAA and BTD21 fluorescence spectra at pH 7.4 and pH 2.3, respectively. The incubated insulin preparations showed fluorescence signals with pFTAA or BTD21, showing that these dyes could be used to assess amyloid formation of insulin preparations (Fig. 2A and B). It should be noted that unincubated insulin detemir (exclusively) showed strong BTD21 fluorescence, possibly due to the fatty acid conjugate of this insulin drug. Furthermore, we examined the increase in pFTAA and BTD21 fluorescence intensity of incubated insulin preparations by subtracting the intensity of unincubated ones (Fig. 2C and D). Interestingly, preparations of insulin glargine I and glargine II showed significantly increased pFTAA fluorescence, indicating that glargine type insulin preparations could form fibril-type amyloids (Fig. 2C). On the other hand, insulin lispro, detemir and glulisine showed increased BTD21 after incubation, suggesting that these insulin preparations form filament-type amyloids (Fig. 2D). Notably, the glargine, lispro, and detemir insulin types had all been used as insulin treatment for patient X and/or Y, whose tissue sections were analyzed in Fig. 1B. Therefore, in the present study, we hereafter focused on these insulin preparations.

**Fig. 3:**
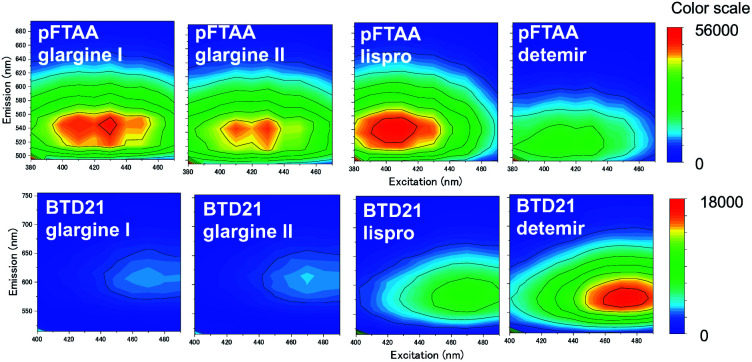
2D plots of excitation/emission pFTAA (upper panel) and BTD21 (lower panel) with selected incubated insulin preparations for clinical use.

The fluorescence property was further confirmed by recording 2D plots of excitation *vs.* emission as shown in Fig. 3. The 2D plots provide sensitive spectral fingerprints of the two forms. As shown in the figure, pFTAA stained glargine I and glargine II, and BTD21-stained lispro and detemir showed similar 2D plots with pFTAA-stained fibrils and BTD21-stained filaments (shown in Fig. S1†), respectively. With pFTAA, however, there is also a strong response for insulin lispro, but the spectral emissions are slightly blue-shifted compared with the spectral 2D features of the fibril binding glargine I and glargine II (Fig. 3). It should be noted that insulin preparations were incubated as is (not at low pH).

For comparison, fluorescence of a conventional amyloid probe, thioflavin T (ThT), for incubated insulin preparations was measured (Fig. S2†). However, the ThT intensities of the incubated insulin preparations were significantly lower than those of insulin fibrils derived from bovine insulin, and only a small increase was detected after incubation. In addition, there was no significant difference between detemir, the glargines and glulisine, for which pFTAA and BTD21 discriminated amyloid types as shown in Fig. 2 and 3. This result strongly suggests that pFTAA and BTD21 are useful for the detection and characterization of the polymorphic amyloid structure of insulin preparations, which is difficult with ThT fluorescence.

### Transmission electron microscopy (TEM) imaging of fibrils and filaments

**Fig. 4:**
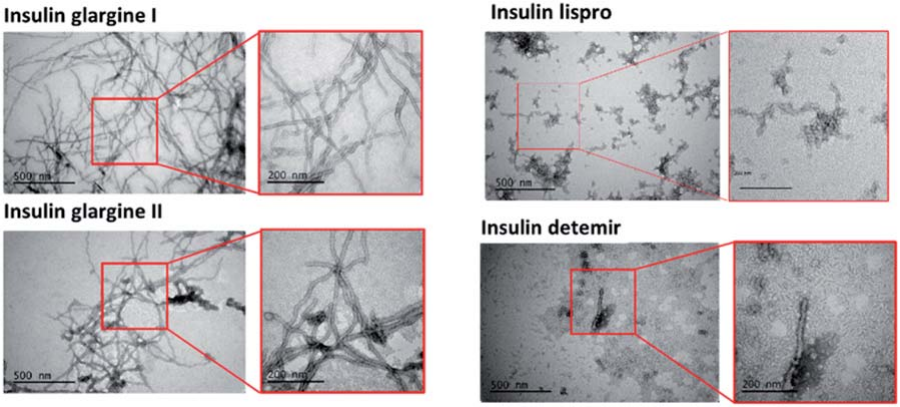
TEM images of incubated insulin preparations (glargine I, glargine II, detemir and lispro).

TEM images were taken to confirm the aggregation of the insulin preparation (Fig. 4). As shown in the figure, pFTAA-positive insulin glargine I and glargine II showed several micrometer long fibrils with a width of 10–15 nm, which is consistent with a previous observation of fibrillar insulin amyloid formation.^6,18^ On the other hand, BTD21-positive insulin lispro and insulin detemir showed morphologically different aggregates: more flexible structures for lispro, and thicker (20 nm wide) less ordered morphology co-localizing with small disordered species for detemir (Fig. 4). Taken together, the results from fluorescence spectroscopy and TEM suggest that pFTAA binds to mature amyloid fibrils with spectral characteristics as was done for glargine I and glargine II, whereas BTD21 binds to less ordered and flexible amyloids.

### Hyperspectral fluorescence images of aggregated insulin preparations

**Fig. 5:**
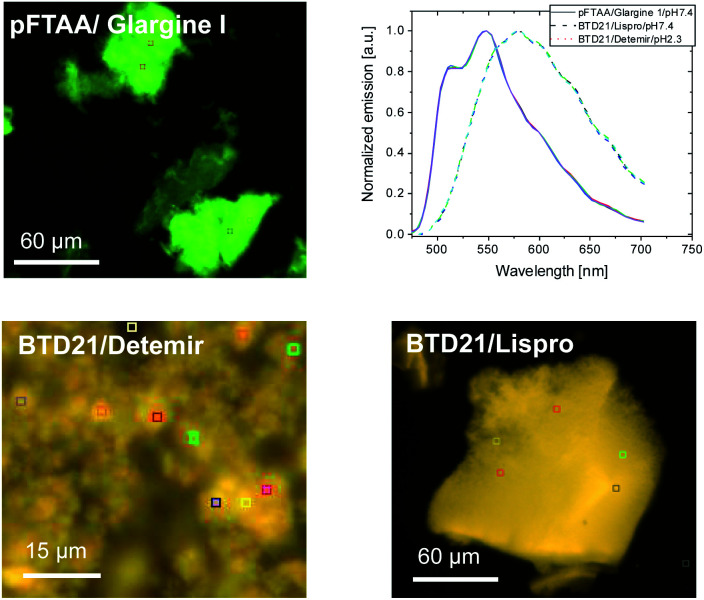
Hyperspectral fluorescence microscopic images of the incubated insulin preparations (glargine I, lispro and detemir). The insulin preparations were stained using pFTAA at pH 7.4 and BTD21 at pH 7.4 (lispro) and pH 2.3 (detemir). The upper right panel shows the normalized fluorescence spectra of five ROIs for each image, indicating a homogeneous spectral distribution in each case.

Because sample inhomogeneity and scatter affects the spectral efficiency when using a plate-reader, the stained insulin preparations were also examined using hyperspectral fluorescence microscopy to reveal structural differences along with their pertinent emissions. For example, it was previously shown that Aβ amyloid polymorphisms were observed in mouse brain tissues with hyperspectral fluorescence microscopy using BTD21.^16^ Representative hyperspectral fluorescence microscope images of aggregated insulin preparations, along with the typical spectral emissions at various places, were taken using glargine I as a pFTAA-positive sample, and lispro and detemir as BTD21-positive samples, respectively (Fig. 5). Both types of insulin preparations were stained by these probes and showed a good agreement with the spectroscopic data (Fig. 2A and B): peaks around at 520 and 550 nm for glargine I stained with pFTAA and a broad emission just below 600 nm for detemir and lispro stained with BTD21. Importantly, there was no significant difference among various places in the aggregates, suggesting that there was structural homogeneity of the aggregates of the insulin preparations.

As a reference, hyperspectral images with spectral emissions are shown for bovine insulin fibrils and filaments using both pFTAA and BTD21 in Fig. S3.† These results confirm previous findings using pFTAA in conjunction with AFM studies of the aggregated bovine insulin.^11^ The obtained image and spectra were similar with the ones for insulin preparations (Fig. 5).

### Cell cytotoxicity of incubated insulin analogues

**Fig. 6:**
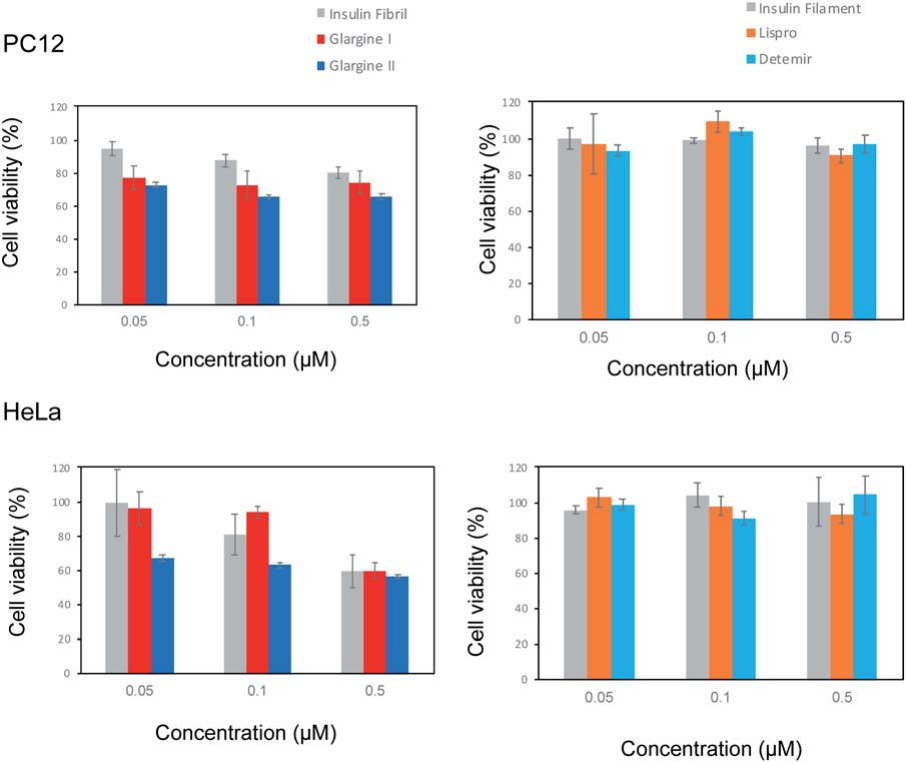
Cytotoxicity of incubated insulin preparations at various concentrations with PC12 and HeLa cells. Cell viability after incubation for 24 h at 20 000 cells per well was evaluated with an MTT assay. Left panels show fibril and pFTAA-positive insulin preparations. Right panels show filament and BDT21-positive insulin preparations.

To investigate the cytotoxic properties of aggregates of incubated insulin preparations, the cell toxicity of insulin glargine I and glargine II as pFTAA-positive samples, and insulin lispro and detemir as BTD21-positive samples was examined by employing an MTT assay with PC12 and HeLa cells, which are commonly used as model cells to assess amyloid toxicity (Fig. 6). Bovine insulin fibrils and filaments were used as the respective control samples of the pFTAA-positive and BTD21-positive insulin samples. Both insulin glargines showed dose-dependent cell toxicity for PC12 and HeLa cells. This result is consistent with the high toxicity of insulin fibrils^6,7^ and with our previous case report.^5^ In contrast, interestingly, insulin lispro and detemir showed no apparent dose-dependence, which was similar with the insulin filaments. This indicates that the aggregates of these insulin preparations were not cytotoxic. This result also agrees well with our previous finding that filamentous insulin amyloids were less cytotoxic.^6,7^ Importantly, taken together with the results of fluorescence spectroscopy and microscopic imaging along with the TEM images, these results support the findings of one possible correlation between pFTAA reactivity and toxic fibrillar aggregation of insulin preparations, and another correlation between BTD21 reactivity and less toxic insulin aggregation with less-ordered structures.

## Conclusions

Insulin-derived amyloidosis (insulin ball) is an iatrogenic complication of diabetic patients treated with insulin drugs. Several variants, which are different in terms of protein sequence and chemical modifications, are combined using long-lasting and rapid uptake insulin preparations to provide an individualized treatment. The inclusions formed locally at the subcutaneous injection site are often called insulin ball and are associated with poor insulin availability, causing complications for dosing to control glucose uptake. This negative effect is mediated by native insulin adsorption to insulin amyloid.^19^ Further pathological issues with insulin ball formation are rather unknown and have only recently been investigated.^5^

In this study, we discovered that several types of amyloid polymorphic aggregates were present in two patients with insulin ball formation using pFTAA and BTD21, showing that these novel amyloid probes could be a powerful tool to analyze insulin balls in patients. Furthermore, 9 insulin preparations were investigated for their propensity to form amyloid aggregates. It was demonstrated that, depending on insulin preparation, two main classes of amyloid aggregates, pFTAA-positive needle-like amyloids and BTD21-positive less-ordered flexible amyloids, were formed in the high temperature condition without buffer exchange to acid buffers. Insulin fibrils such as those formed by insulin glargine are cytotoxic to cells in culture whereas less-ordered flexible insulin amyloids appear non-toxic. Insulin amyloid aggregates could be formed in a patient's daily life during insulin storage or at the sites of injection. Our results emphasize that the chronic pathological effect of iatrogenic insulin amyloid fibrils comprising amyloidomas should be further investigated.

## Author contributions

T. Z. conceived the project. T. Z. and M. L. designed the experiments. K. Y. and M. L. performed all the *in vitro* fluorescent experiments. J. Z. and P. H. synthesized fluorescent probes. K. Y., W. M. and R. K. performed toxicity experiments. P. H. carried out TEM observation. S. N., M. L., T. N. and K. I. performed hyperspectral imaging. K. Y. and M. L. and T. Z. wrote the initial manuscript draft, and all the authors discussed the results and contributed to the manuscript preparation.

## Conflicts of interest

There are no conflicts to declare.

## Supplementary Material

RA-010-D0RA07742A-s001
