# Supplementary material for: Insulin amyloid polymorphs: implications for iatrogenic cytotoxicity
Source: RSC Adv. 2020 Oct 12;10(62):37721–7. doi: 10.1039/d0ra07742a (PMC9057202; doi:10.1039/d0ra07742a)
Supplement: RA-010-D0RA07742A-s001 [file RA-010-D0RA07742A-s001.pdf]

Electronic Supplementary Material (ESI) for RSC Advances.
This journal is © The Royal Society of Chemistry 2020

Supplementary Information

Insulin Amyloid Polymorphs: Implications for Iatrogenic Cytotoxicity

Keisuke Yuzu,[†a] Mikael Lindgren,[†b] Sofie Nyström,[c] Jun Zhang,[c] Wakako Mori,[a] Risako Kunitomi,[a] Terumasa Nagase,[d] Keiichi Iwaya,[e] Per Hammarström[c] and Tamotsu Zako[*a]

[a.] *Department of Chemistry and Biology, Graduate School of Science and Engineering, Ehime University, 2-5, Bunkyo-cho, Matsuyama, Ehime 790-8577 Japan. E-mail: zako.tamotsu.us@ehime-u.ac.jp*
[b.] *Department of Physics, Faculty of Natural Sciences, Norwegian University of Science and Technology, NO-7491 Trondheim, Norway.*
[c.] *IFM Chemstry, Linköping University, SE-58183 Linköping, Sweden*
[d.] *Department of Metabolism and Endocrinology, Tokyo Medical University Ibaraki Medical Center, Ibaraki 300-0395, Japan*
[e.] *Department of Pathology, SASAKI Institute, Kyoundo Hospital, Tokyo 101-0062 Japan*
† Equal contribution.

Table of Contents

Fig. S1 Binding of pFTAA and BTD21 to insulin fibrils and filaments.

Fig. S2 ThT fluorescence of incubated insulin preparations with insulin fibrils.

Fig. S3 Hyperspectral fluorescence images of stained *in vitro*-formed bovine insulin fibrils and filaments.

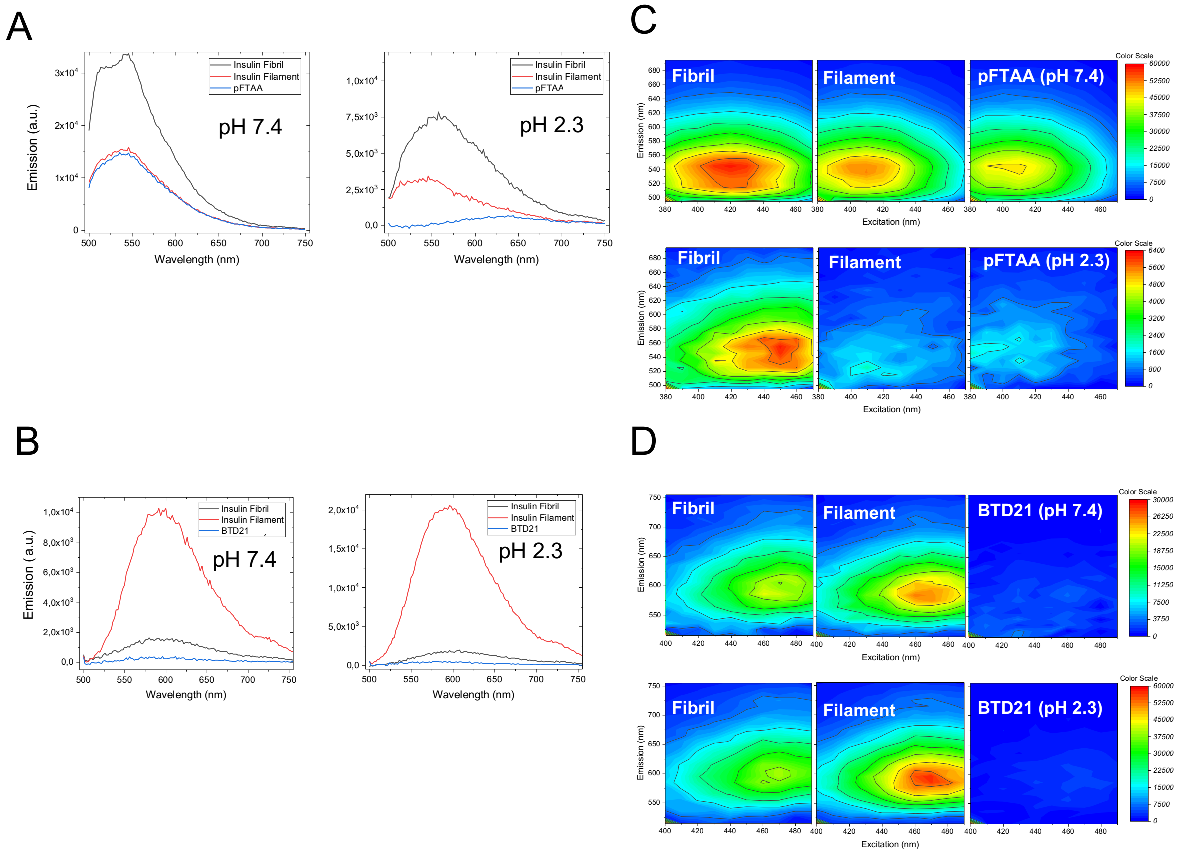

Figure S1 Binding of pFTAA and BTD21 to insulin fibrils and filaments. (A, B) Fluorescence spectra of bovine insulin fibrils and filaments using A) pFTAA and B) BTD21 amyloid probe at pH 7.4 and pH 2.3, respectively. The excitation wavelength was 470 nm (slits 10/5, ex/em). Note that the amplitude scales are different although the settings are the same. (C, D) 2D scan/3D plots of the excitation/emission of C) pFTAA and D) BTD-21 with insulin fibrils and filaments at pH 7.4 (top rows) and pH 2.3 (lower rows). To compare the intensities, the amplitude scales are the same along each row.

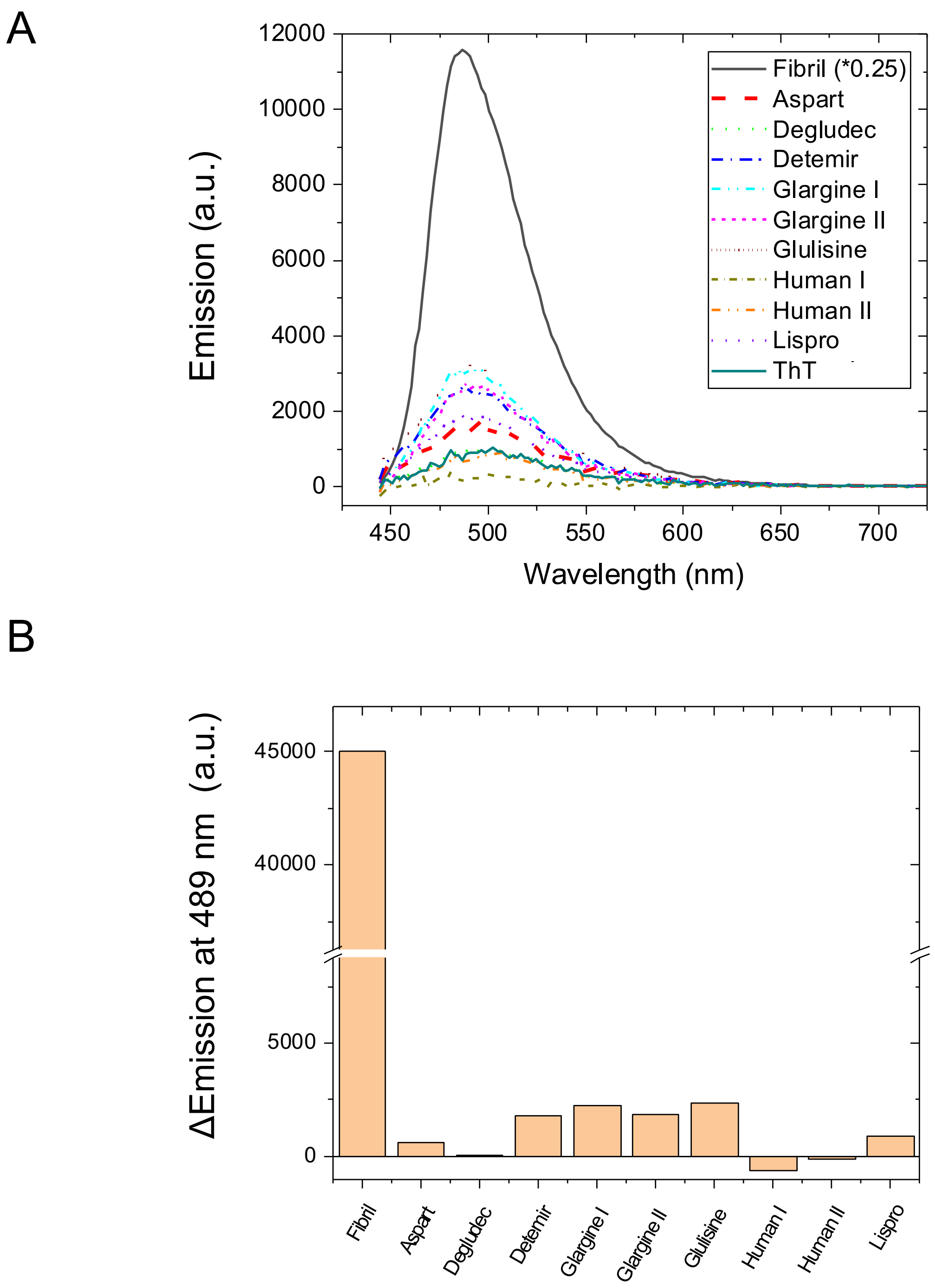

Figure S2 ThT fluorescence of incubated insulin preparations with insulin fibrils shown for comparison. A) Spectral raw data. Note that the spectrum of ThT multiplied with 0.25 was shown for comparison of all spectras. B) The intensity at 489 nm with the intensity of the blank (ThT) sample subtracted.

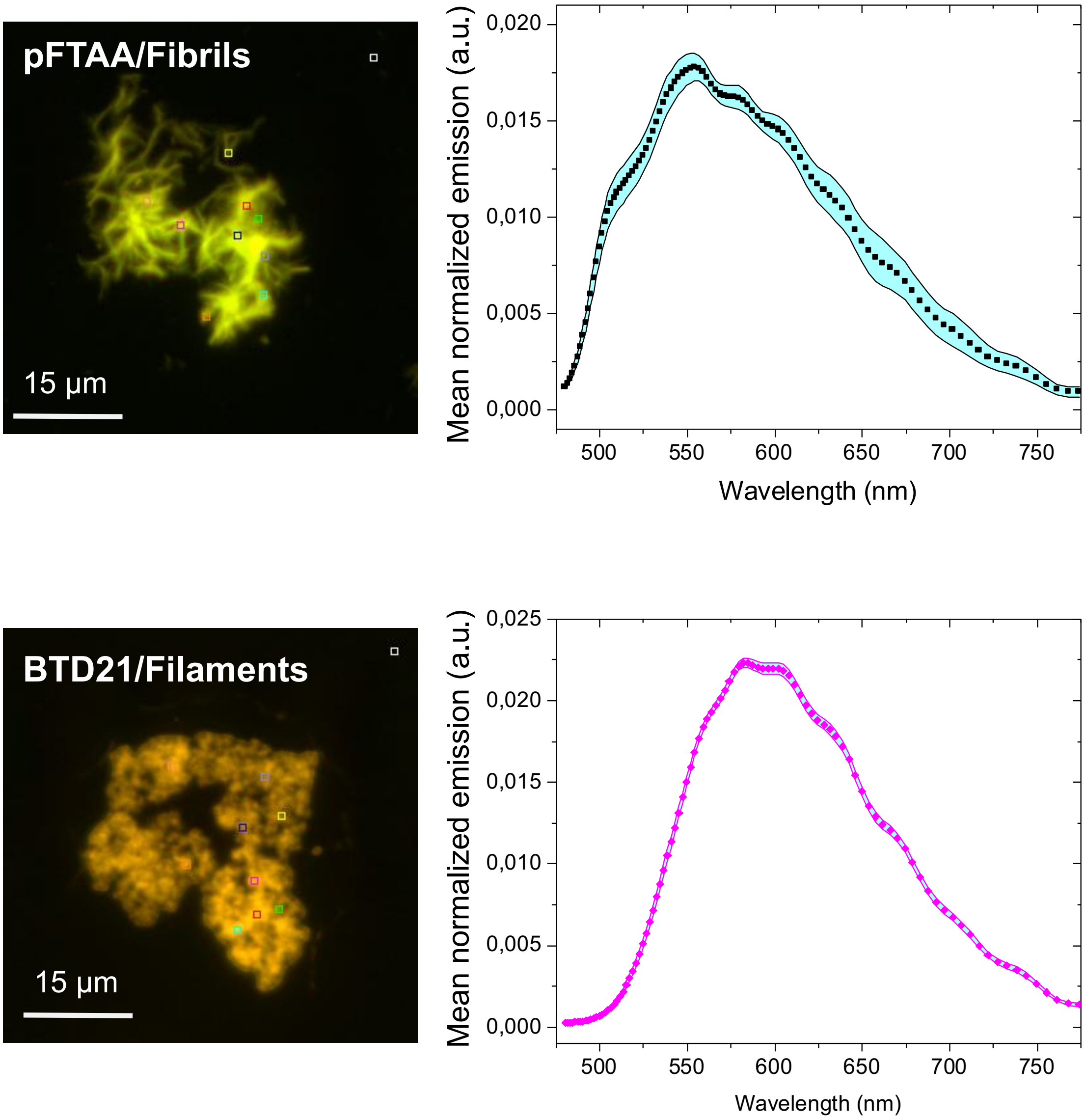

Figure S3 Hyperspectral fluorescence images of stained *in vitro*-formed bovine insulin fibrils and filaments. The top panel shows fibrils stained with pFTAA. The lower panel shows filaments stained with BTD21. The excitation wavelength was 436 nm. To calculate the corresponding spectra (right panels), spectra of nine ROIs were first normalized to the area, then the average spectrum along with the standard deviation for each wavelength was calculated. The symbols represent the average spectrum and the bands surrounding the symbols indicated the variation of the normalized spectra in terms of the calculated standard deviation of the nine normalized components.
